# *Bipolaris sorokiniana*-Induced Black Point, Common Root Rot, and Spot Blotch Diseases of Wheat: A Review

**DOI:** 10.3389/fcimb.2021.584899

**Published:** 2021-03-11

**Authors:** Abdullah M. Al-Sadi

**Affiliations:** Department of Plant Sciences, College of Agricultural and Marine Sciences, Sultan Qaboos University, Alkhoud, Oman

**Keywords:** *Triticum aestivum*, *Helminthosporium*, *Cochliobolus sativus*, *Drechslera*, control

## Abstract

Wheat is among the ten top and most widely grown crops in the world. Several diseases cause losses in wheat production in different parts of the world. *Bipolaris sorokiniana* (teleomorph, *Cochliobolus sativus*) is one of the wheat pathogens that can attack all wheat parts, including seeds, roots, shoots, and leaves. Black point, root rot, crown rot and spot blotch are the main diseases caused by *B. sorokiniana* in wheat. Seed infection by *B. sorokiniana* can result in black point disease, reducing seed quality and seed germination and is considered a main source of inoculum for diseases such as common root rot and spot blotch. Root rot and crown rot diseases, which result from soil-borne or seed-borne inoculum, can result in yield losses in wheat. Spot blotch disease affects wheat in different parts of the world and cause significant losses in grain yield. This review paper summarizes the latest findings on *B. sorokiniana*, with a specific emphasis on management using genetic, chemical, cultural, and biological control measures.

## Introduction

Wheat (*Triticum aestivum*) is among the most widely cultivated crops in the world. Wheat production exceeded 734 million tons in 2018 from 214 million ha of land (FAO, 2021). China, India, Russia, USA, and France were the largest producers of wheat in the world in 2018, accounting for more than 50% of the world’s production (FAO, 2021).

Wheat production is limited by several biotic stresses, with diseases being a major limiting factor to wheat production worldwide. The total number of wheat diseases exceeds 200, but 50 diseases cause economic losses and are widely distributed (Wiese, 1987; Al-Sadi, 2016; Jarroudi et al., 2017; Lalic et al., 2017; Riaz et al., 2017; Sharma et al., 2017). Each year about 20% of wheat is lost due to diseases. Some of the major wheat diseases are rusts, spot blotch, common root rot, smut, tan spot, Septoria blotch, powdery mildew, fusarium head blight, blast and a number of viral, nematode, and bacterial diseases (Wiese, 1987; Chowdhury et al., 2013; Fetch et al., 2015; Zhu et al., 2015; Al-Sadi, 2017; Abdullah et al., 2020; Aboukhaddour et al., 2020; Gultyaeva et al., 2020). They can reduce yield or result in mortality of the infected plants. The focus of this review will be on the etiology and management of *B. sorokiniana* diseases in wheat.

The genus *Helminthosporium* is a large group of the class Hyphomycetes that includes many species pathogenic to plants and animals. This genus has been split into three genera: *Exserohilum, Bipolaris*, and *Drechslera*, on the basis of conidial ontogeny and morphology (Alcorn, 1988).

## *Bipolaris sorokiniana*

*B. sorokiniana* (Sacc.) Shoemaker, (syn. *Helminthosporium sativum* Pammel, King & Bakke, *H. sorokinianum* Sacc. in Sorokin, and *Drechslera sorokiniana* (Sacc.) Subramanian & Jain, causes diseases on a number of cereals, including wheat (Tunali et al., 2008; Devi et al., 2018; Gultyaeva et al., 2018; Gupta et al., 2018b; Jamil et al., 2018; Singh et al., 2019; Villa-Rodríguez et al., 2019; Li et al., 2020). The teleomorph for this fungus is *Cochliobolus sativus* (Ito & Kuribayashi) Drechs. ex Dastur, which is the sexual (perfect) state. *C. sativus* was not reported in nature, except in Zambia (Raemaekers, 1991). However, sexual reproduction of *C. sativus* has been rarely reported (Sultana et al., 2018). On the other hand, most of the reproduction of *B. sorokiniana* occurs through the production of asexual conidia (Gupta et al., 2018a).

**Figure 1:**
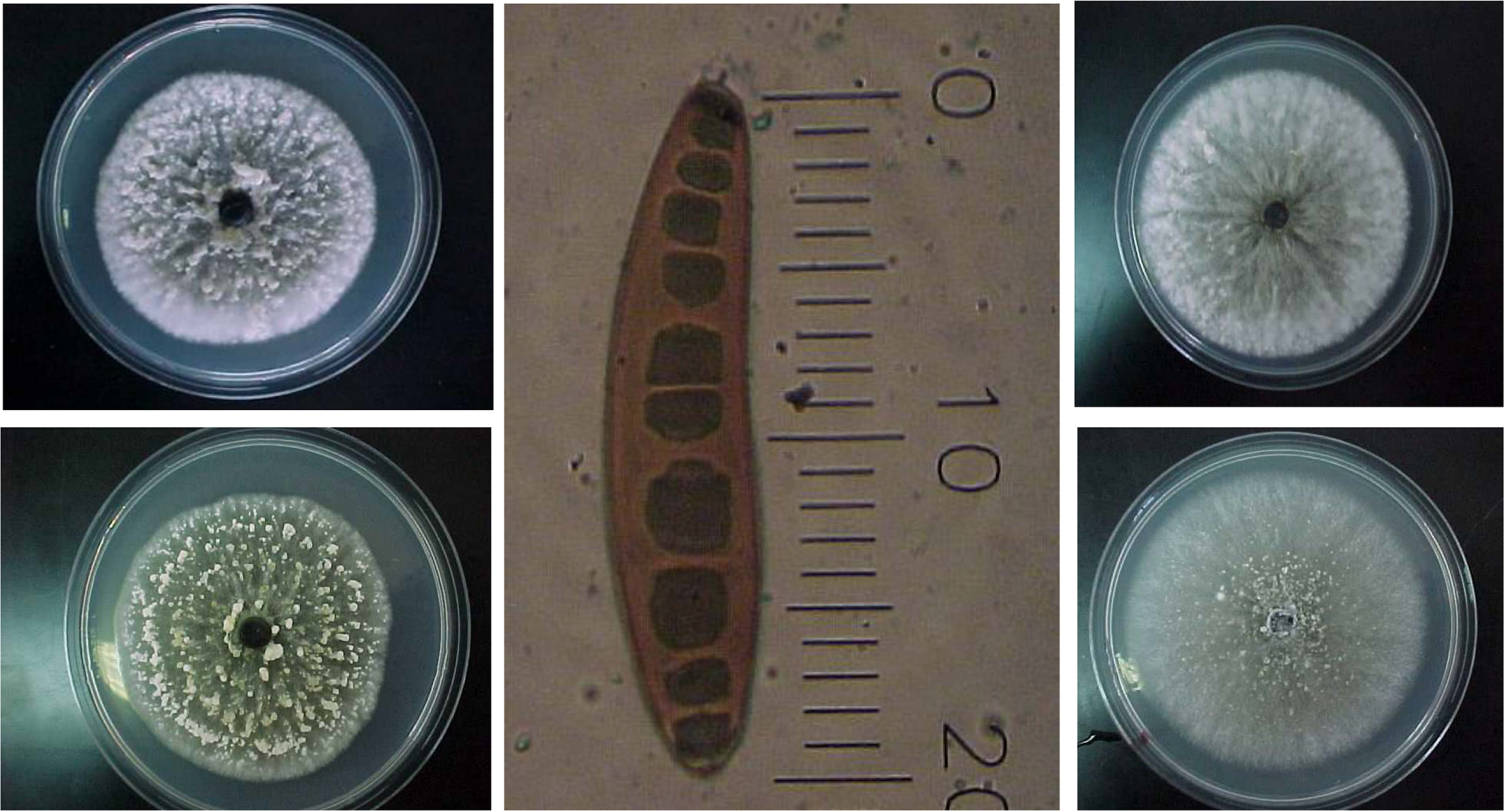
Morphology of *Bipolaris sorokiniana* culture and spores (1 scale is equivalent to 5 µm) grown on potato dextrose agar. The mycelial growth of the four isolates shows mixed color (white and black) as explanied by Navathe et al. (2020), with varying intensities of the black color among the isolates.

The genus *Bipolaris* has brown conidiophores, mostly simple, producing conidia through the apical pore. The conidia are brown, several-celled (phragmosporous), elliptical, straight, or curved, germinating by one germ tube at each end (Barnett and Hunter, 1998; Navathe et al., 2020) (**Figure 1**). *B. sorokiniana* has olive-brown, ovate conidia, with tapered ends and a prominent basal scar. The conidia are 15-28 X 40-120 µm and have 3- to 10- septa (Wiese, 1987) (**Figure 1**).

## Diseases Caused by *B. sorokiniana*

*Bipolaris sorokiniana* attacks different cereals, including wheat, and causes common root rot, spot blotch, and black point diseases. Root rot is one of the most widespread diseases of wheat and it occurs in all areas where wheat is grown. Losses in wheat due to common root rot and seedling blight vary. Canada lost approx. 5.7% of wheat during 1969–1971 due to common root rot, which is equivalent to $42 million (Ledingham et al., 1973). Smiley et al. (2005) estimated 35% loss in wheat yield due to crown rot in the Pacific Northwest. Spot blotch is found wherever wheat is grown, and it can cause significant losses (15-25%) in warm areas (Gupta et al., 2018a). Seed infection by *B*. *sorokiniana* can result in black point disease, which may result in root rot and seedling blight (Wiese, 1987; Al-Sadi and Deadman, 2010; Li et al., 2019b).

*B. sorokiniana* attacks several host plants from different genera and families (Wiese, 1987; Farr et al., 1989). The major plant hosts (listed by the genera name) that attacked by *B. sorokiniana* are *Agrohordeum, Agropyron, Agrostis, Ammophila, Andropogon, Arthraxon, Avena, Bouteloua, Bromus, Buchloe, Calamagrostis, Calamovilfa, Cenchrus, Chloris, Cynodon, Dactylis, Dendrobium, Dichanthelium, Digitaria, Echinochloa, Elymus, Eragrostis, Eremopyrum, Festuca, Hordeum, Hystrix, Koeleria, Linum, Lolium, Medicago, Muhlenbergia, Oryzopsis, Panium, Phalaris, Phleum, Poa, Secale, Setaria, Sorghum, Stipa, Trifolium, Triticum, Vulpia, Zea*, and *Zizania* species (Farr et al., 1989). Disease symptoms and yield losses in these hosts are variable. *B. sorokiniana* do not have host specialization (forma speciales). However, isolates have been found to differ in their aggressiveness on wheat and barley (Al-Sadi, 2016).

## Black Point

### Importance and Etiology of Black Point

Black point is a disease of cereal seeds, exhibiting a brown to black tip at the embryo end of the grain. The affected kernels usually become heavier than normal. The disease can result in lowering quality and market value of grains, production of fungal toxins in the seeds that may become harmful to livestock, and causing seedling blight, root rot and different diseases. In addition, it can reduce seed germination, seedling emergence, total photosynthetic area, and normal growth of plants (Al-Sadi and Deadman, 2010; Neupane et al., 2010; Ghosh et al., 2018; Li et al., 2019b; Somani et al., 2019).

The disease is caused by *B. sorokiniana* (Sisterna and Sarandon, 1996; Xu et al., 2018b; Li et al., 2019b; Somani et al., 2019; Li et al., 2020). In addition, some reports indicated the association of *Alternaria alternata*, *Fusarium* spp., and *Penicillium* spp. with wheat seeds developing black point symptoms (Al-Sadi and Deadman, 2010; Gannibal, 2018; Ghosh et al., 2018; Xu et al., 2018b; Li et al., 2019b; Li et al., 2020). Black point has been reported in different parts of the world, including China, Argentina, Oman, Australia, India, and Bangladesh (Rashid et al., 1992; Sisterna and Sarandon, 2005; Al-Sadi and Deadman, 2010; Poole et al., 2015; Xu et al., 2018b).

*Bipolaris sorokiniana* has been reported in the embryo (Weniger, 1923) and in the endosperm of wheat seeds (Rashid et al., 1997). Seed infection in wheat increases after flowering (Andersen, 1952). It can also be affected by the genotype, location (especially in warm and humid climates) and the management practices (Zhang et al., 1990). Penetration into the seed is achieved through the ovary wall and seed coat (Han et al., 2010; Ansari et al., 2017). *B. sorokiniana* was reported to remain viable for 10 years in wheat seeds (Machacek and Wallace, 1952). The fungus can also survive as a resting mycelium for 5 years (Mead, 1942). *B. sorokiniana* is very frequently isolated from the seeds of wheat, reaching as high as 80%–90.5%, and with a common level of infection of about 9%–22%, depending on the cultivar and the prevailing conditions (Rashid et al., 1992; Rashid et al., 1997; Rashid, 1998; Li et al., 2019b).

The incidence of black point disease is affected by many factors, most importantly temperature and humidity. Higher humidity (especially above 90%), rain and relatively lower temperatures (<30 ^o^C) after heading usually increase the disease incidence (Cromey and Mulholland, 1988; Li et al., 2019a; Li et al., 2019b).

### Management of Black Point

Control of *B. sorokiniana* in the seeds of wheat could be achieved through the use of resistant cultivars, fungicides, seed treatment, or biocontrol agents. Cultivars can react differently to seed infection due to several factors such as non-compatibility to infection, restricted pathogen invasion of the seed parts due to inhibitors, or reduced testa permeability (Gannibal, 2018; Singh et al., 2019). In a study by Li et al. (2014) on 403 wheat genotypes in the North China Plain, 62.5% of the genotypes were classified as susceptible, while 37.5% were resistant to black point disease. In another study, considerable variation was found among wheat cultivars in their resistance to black point disease, with no relationship between the earliness of ripening and resistance (Cromey and Mulholland, 1988). Connert and Davidson (1988) showed that wheat cultivar resistance to black point disease can be affected by the causal agent, with some cultivars having more resistance to *B. sorokiniana* than to *A. alternata*.

A study in Pakistan revealed that tebuconazole + imidacloprid and difenoconazole + cyproconazole were the most effective chemicals for the management of black point disease of wheat (Shahbaz et al., 2018). Triazole fungicides (e.g., propiconazole and tebuconazole) inhibit the synthesis of sterols, which are building blocks of the membranes of fungal cells. This makes them ideal chemicals for the management of *Bipolaris* and other fungal pathogens (Ansari et al., 2017; Somani et al., 2019). Treating seeds with fungicides helps protect wheat seeds from infection. In addition, it helps manage diseases associated with seed infection, including root and crown rot. Some of the common fungicides used in seed treatment include fludioxonil and difenoconazole (Wei et al., 2021) and Vitavax-200 (Carboxin 37.5% + Thiram 37.5%) and Homai-80WP (Thiophanate methyl 50% + Thiram 30%) (Malaker and Mian, 2009).

The use of biocontrol agents has been effective in reducing black point disease. *Bacillus amyloliquefacien*s, *B. megaterium, Trichoderma harzianum*, and *Epicoccum* sp. were found antagonistic against the causal agents of black point disease (El-Gremi et al., 2017). The isolates also improved germination and seedling growth of wheat, with *B*. *amyloliquefacien*s being the most efficacious, as it was as effective as the fungicide diniconazole in increasing the weight of kennels. In another study, the antifungal compounds produced by *B. vallismortis* were effective in inhibiting black point fungi (Kaur et al., 2015; Kaur et al., 2017). A study by Mónaco et al. (2004) showed that *T. harzianum* and *T. koningii* significantly inhibited the growth and caused mycelial abnormalities in *Bipolaris sorokiniana* and *A. alternata*.

## Spot Blotch

### Importance and Etiology of Spot Blotch

Spot blotch is a common disease on wheat in all continents (Duveiller et al., 1998; Al-Saadi et al., 2002; Neupane et al., 2010; Al-Sadi, 2016; Devi et al., 2018; Gultyaeva et al., 2018; Gupta et al., 2018a; Gupta et al., 2018b). Losses due to spot blotch are high, especially in warmer areas of the world. They have been reported to reach 16%–43% (Sharma and Dubin, 1996; Duveiller and Sharma, 2009; Ayana et al., 2018; Devi et al., 2018). In addition, the hotspot for spot blotch disease is in South Asia (Van Ginkel and Rajaram, 1998; Joshi et al., 2007; Sharma et al., 2018; Sultana et al., 2018).

Spot blotch symptoms appear as brown lesions with yellow halos, which enlarge with time to cover larger areas of the leaf. Lesions can turn olive brown in color, especially under humid conditions that promote sporulation of the fungus (Al-Sadi, 2016; Gupta et al., 2018a; Gupta et al., 2018b). *Bipolaris sorokiniana* is the pathogen responsible for spot blotch disease in wheat (Devi et al., 2018; Gupta et al., 2018a; Gupta et al., 2018b; Tembo et al., 2018; Aggarwal et al., 2019). The symptoms of *Pyrenophora tritici-repentis*-induced tan spot, and Alternaria leaf blight resemble those of spot blotch. One difference is that tan spot is characterized by the appearance of dark fruiting structures, called pseudothecia, on wheat straw, which is not the case for spot blotch (Carmona et al., 2006). Spot blotch differs from Alternaria blight by the development of dark spot areas, which represent masses of conidia that are produced at later infection stages (Neupane et al., 2010; Viani et al., 2017). The spot blotch symptoms elongate and coalesce (Chand et al., 2010).

Leaf infection by *B. sorokiniana* could come from seeds, root or air. If the pathogen is in the soil, then infection could occur through stomata on the hypocotyl, from where the fungus progresses to the root, shoot and coleoptile (Sprague, 1950). Spore germination can occur within 4–6 h and penetration by *B. sorokiniana* occurs through stomata and epidermis (Raguchander et al., 1988).

Studies in India and Brazil have shown that spot blotch is usually favored by warm weather (Chaurasia et al., 2000; Kumar et al., 2002; Acharya et al., 2011; Singh, 2017). Also, high humidity is an important factor in enhancing symptom development (Viani et al., 2017). Infection usually starts on the older leaves (Gupta et al., 2018a). In addition, water stress and terminal heat stress have negative effects on the resistance of wheat to *B. sorokiniana* (Duveiller and Sharma, 2009).

### Management of Spot Blotch

No complete resistance to spot blotch has been reported in wheat, but wheat cultivars have been reported to differ in resistance to the disease (**Table 1**) (Ahirwar et al., 2018; Ayana et al., 2018; Gurjar et al., 2018; Jamil et al., 2018; Singh et al., 2018; Tembo et al., 2018). Therefore, breeding and selecting resistant cultivars is the best option for managing spot blotch in the long term (Gupta et al., 2018a). Among 150 wheat genotypes screened in Zambia, the genotypes 19HRWSN6, 19HRWSN7, and 19HRWSN15 were found resistant (Tembo et al., 2018). In addition, a study on 60 wheat genotypes in Nepal indicated that the genotype NL750 had a high level of resistance to spot blotch, while the tolerant genotype BL1473 is able to produce good yields despite the high disease levels (Sharma et al., 2004; Rosyara et al., 2007).

**Table 1:** Examples of wheat genotypes having less susceptibility to spot blotch.

Country	Wheat genotypes/cultivars	References
Afganistan	PAMIR‐94	(Bainsla et al., 2020)
Brazil	BH 1146	(Singh et al., 2016; Singh et al., 2018)
China	Ning 9415, Ning 8201	(Schlegel, 1997; Bainsla et al., 2020)
India	Chirya 7, Chirya 3, Ning 8139, Suzhou, Milan-3, HD 2888, HD 2967, WR 95, IC529962 and IC443652	(Gurjar et al., 2018; Kumari et al., 2018; Choudhary et al., 2019)
Mexico	BARTAI, WUYA	(Singh et al., 2018)
Nepal	NL750	(Sharma et al., 2004; Rosyara et al., 2007)
Zambia	19HRWSN6, 19HRWSN7 and 19HRWSN15	(Tembo et al., 2018)

Resistance can be induced using some microorganisms and compounds. The combined application of *Trichoderma harzianum* and methyl jasmonate was found to enhance the activities of defense related enzymes, including catalase, ascorbate peroxidase, phenylalanine lyase, and peroxidase (Singh et al., 2019). In addition, methyl jasmonate is known to inhibit spore germination in *B. sorokiniana*. In another study, wheat was found to strongly elicit salicylic acid signaling, followed by an enhanced expression of phenylpropanoid pathway genes, which leads to the accumulation of phenolics that play a role in the resistance against spot blotch (Sahu et al., 2016). Also (Sharma et al., 2018) showed that salicylic acid and syringic acid negatively correlated with spot blotch severity, indicating their role in disease defense.

In a study on the efficacy of 195 bacterial strains in suppressing *B. sorokiniana*, *Bacillus subtilis* TE3 strain proved to be the most efficacious in suppressing the disease (Villa-Rodríguez et al., 2019). The mechanisms of actions of the antagonistic bacterial strain were through colonizing the wheat phyllosphere and the antimicrobial compounds produced by the bacterium. Additionally, *B. safensis* and *Ochrobactrum pseudogrignonense* have been reported to promote resistance to spot blotch in wheat (Sarkar et al., 2018). However, the efficacy of biocontrol agents is usually limited by environmental factors and growing conditions.

Several fungicides have been developed and used for the management of spot botch. The yield increase in fungicide treated plots suffering from leaf diseases compared to untreated plots was 10% in Sweden (Djurle et al., 2018) and 30% in Argentina (Castro et al., 2018). The fungicides carbendazim (Yadav et al., 2013), difenoconazole  (Ishikawa et al., 2012), propiconazole (Singh and Singh, 2007; Gupta et al., 2017), and Azoxistrobin (Navathe et al., 2019) were efficacious in managing spot blotch. In addition, Mishra et al. (2014) showed that silver nanoparticles act as a fungicide against spot blotch. The use of silicon was also found to improve resistance of wheat leaves to *B. sorokiniana* infection (Domiciano et al., 2010). In addition to these management strategies, balanced nutrition and crop rotation should form a part of the integrated management strategies in managing spot blotch in wheat (Sharma et al., 2005; Sharma et al., 2006; Yadav et al., 2013; Mazzilli et al., 2016; Bankina et al., 2018; Sˇvarta and Bimsˇteine, 2019). The application of nitrogen alone without phosphorus and potassium is known to increase the severity of spot blotch (Singh et al., 2012).

## Common Root Rot and Crown Rot Diseases

### Importance and Symptoms

Common root rot and crown rot of wheat are important diseases in most wheat-growing countries, including China, Australia, Middle East, and Europe (Fedel-Moen and Harris, 1987; Tunali et al., 2008; Al-Sadi and Deadman, 2010; Poole et al., 2015; Gupta et al., 2018b; Xu et al., 2018a). They are characterized by the development of necrotic lesions on the roots, subcrown, and crown. The lesions are dark brown to black in color. Development of symptoms on the root is usually followed by symptoms on wheat crowns (Al-Sadi and Deadman, 2010; Qostal et al., 2019).

The disease is caused by *B. sorokiniana* (Tunali et al., 2008; Xu et al., 2018a; Yue et al., 2018), which is also associated with other fungi including *Fusarium pseudograminearum, F. culmorum, Microdochium nivale*, *Pythium* spp., and *Rhizoctonia cerealis* (Moya-Elizondo et al., 2011; Saremi and Saremi, 2013; Kazan and Gardiner, 2018; Xu et al., 2018a; White et al., 2019).

Yield and quality of wheat could be reduced by common root rot and crown rot. Common root rot was reported to result in yield losses of 6%–24% (Wildermuth et al., 1992). Yield reduction due to crown rot has been estimated to range from 0 to 89% in New South Wales, Australia (Klein et al., 1991). In Queensland (Australia), crown rot caused up to 26% yield loss in some fields, with an overall reduction by 5% for the whole state (Burgess et al., 1981), while a reduction by up to 35% was reported in the Pacific Northwest, North America (Smiley et al., 2005). Reduction in yield is usually because of the effect of common root rot and crown rot diseases on the number of tillers and on the number and size of kernels (Duczek and Jones-Flory, 1993).

Common root rot is a disease of dry and warm areas. Disease severity and incidence is affected by soil moisture, soil temperature, cultural practices, pathogen population in the soil, and time of infection. Disease severity increases when the plant is under stress or grown in warm soil and less moisture (Mathieson et al., 1990; Acharya et al., 2011). In addition, the incidence of common root rot was found to be affected by the soil populations of *B. sorokiniana* at the time of planting (Boer et al., 1991). Propagules of *B. sorokiniana* can go to a depth of 40 cm in the soil, but the population of the fungus is highest in the top 10 cm (Mathieson et al., 1990).

### Management of Common Root Rot and Crown Rot

Different methods have been used in the control of common root rot and crown rot of wheat. The use of the endophytic bacterium *Pseudomonas mediterranea* resulted in a significant reduction in root and crown rot of wheat in Pakistan (Ullah et al., 2020). Disease severity index of wheat common root rot decreased from 90.8% to 27.7% following the use of the actinobacterium *Nocardiopsis dassonvillei* as a biocontrol agent, which was attributed to the ability of this isolate to produce siderophores and hydrogen cyanide (Allali et al., 2019). The actinobacterium was also found to enhance growth of wheat through the production of indole-3-acetic acid. In another study, the bacterial strain *Lysobacter enzymogenes* C3 and the fungal strain *Rhizoctonia* BNR-8-2 were found to result in a significant reduction in the common root rot of wheat, which was attributed to the production of chitinases, β-1,3-glucanases and antibiotics, especially by *L. enzymogenes* C3 (Eken and Yuen, 2014). Yue et al. (2018) showed that the biocontrol fungus *Chaetomium globosum* is effective in inhibiting *B. sorokiniana* associated with wheat common root rot, which is attributed to the production of secondary metabolites by *C. globosum*.

Cultural practices are important for the management of plant diseases. Crop rotation of wheat with *Brassica carinata* was found to result in a significant reduction in common root rot and crown rot diseases (Campanella et al., 2020). In Iran, soil solarization was found effective in reducing wheat root rot (Saremi and Saremi, 2013). The use of organic agriculture helped reduce populations of *Fusarium* populations associated with crown rot of wheat in Canada (Fernandez et al., 2011), while zero tillage was found to increase wheat yields and reduce the incidence of wheat root rot in Mexico (Govaerts et al., 2006).

Different cultivars of wheat were reported to differ in resistance to common root rot (Al-Sadi and Deadman, 2010; Manghwar et al., 2018). In addition, the production of GmPGIP3 transgenic wheat plants enhanced the resistance of wheat to *Bipolaris sorokiniana*-induced common root rot as well as *Gaeumannomyces graminis* var. *tritici*-induced take-all diseases in wheat (Wang et al., 2015). Fungicides are not a good choice for the management of wheat root and crown diseases (Fernandez et al., 2010).

## Conclusion

*Bipolaris sorokiniana* is a serious pathogen, not only because it results in significant yield losses, but also because it can attack most wheat organs, including roots, crown area, stems, leaves and kernels. This means that management strategies should not only focus on limiting the presence of the fungus in the aerial parts of the plants, but attention should be given to *B. sorokiniana* inoculum present in soil. In addition, it is important to develop an integrated disease management program for managing *B. sorokiniana* using cultural practices, biological control and chemical fungicides. Since the search for biocontrol agents has been given more attention during recent years, it is important to find antagonistic strains that can complement cultural and chemical practices in the field. The search for new sources for resistance should consider finding less susceptible cultivars to all diseases caused by *B. sorokiniana*, instead of focusing on one disease.

## Author Contributions

The author confirms being the sole contributor of this work and has approved it for publication.

## Funding

Thanks to the financial support from Sultan Qaboos University, VALE Oman and Oman Animal and Plant Genetic Resources Center.

## Conflict of Interest

The author declares that the research was conducted in the absence of any commercial or financial relationships that could be construed as a potential conflict of interest.
